# Dipraseodymium(III) pyroborate molybdate(VI), Pr_2_(B_2_O_5_)(MoO_4_)

**DOI:** 10.1107/S1600536808010386

**Published:** 2008-05-03

**Authors:** Peter Held, Petra Becker

**Affiliations:** a Institut für Kristallographie, Universität zu Köln, Zülpicher Strasse 49b, D-50674 Köln, Germany

## Abstract

Single crystals of triclinic Pr_2_(B_2_O_5_)(MoO_4_) were prepared from an incongruently melting flux in the system Pr_2_O_3_–MoO_3_–B_2_O_3_ in a platinum crucible in an atmosphere of air. In the crystal structure, distorted edge-sharing [PrO_8_] square anti­prisms form a three-dimensional framework. These are further linked by isolated [MoO_4_] tetra­hedra and isolated pyroborate groups [B_2_O_5_], the latter consisting of two independent trigonal [BO_3_] groups sharing one O atom. The [MoO_4_] tetra­hedra and the [B_2_O_5_] groups are arranged in alternating layers parallel to the *ab* plane.

## Related literature

A rough investigation of the ternary systems *RE*_2_O_3_—B_2_O_3_—MoO_3_ (*RE* = rare earth element) has been reported by Lysanova *et al.* (1983 ▶) and Dzhurinskii & Lysanova (1998 ▶). X-ray powder diffraction data of *RE*_2_(B_2_O_5_)(MoO_4_) compounds with *RE* = Pr, Nd, Sm, Eu, Gd and Tb were reported by Lysanova *et al.* (1983 ▶). Geometric parameters of [BO_3_] groups were reviewed by Zobetz (1982 ▶).

## Experimental

### 

#### Crystal data

Pr_2_(B_2_O_5_)(MoO_4_)
*M* *_r_* = 543.38
Triclinic, 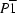

*a* = 5.2806 (5) Å
*b* = 7.0278 (5) Å
*c* = 10.5824 (9) Å
α = 74.557 (6)°
β = 76.307 (7)°
γ = 73.065 (6)°
*V* = 356.69 (5) Å^3^
*Z* = 2
Mo *K*α radiation
μ = 15.20 mm^−1^
*T* = 291 (2) K
0.20 × 0.15 × 0.12 mm

#### Data collection

Enraf–Nonius CAD-4 diffractometer
Absorption correction: ψ scan (*MolEN*; Fair, 1990 ▶) *T*_min_ = 0.296, *T*_max_ = 0.999 (expected range = 0.048–0.161)
4733 measured reflections
2155 independent reflections
1983 reflections with *I* > 2σ(*I*)
*R*_int_ = 0.019
3 standard reflections every 100 reflections intensity decay: 1.7%

#### Refinement

*R*[*F* ^2^ > 2σ(*F* ^2^)] = 0.031
*wR*(*F* ^2^) = 0.082
*S* = 1.13
2155 reflections
127 parameters
Δρ_max_ = 2.55 e Å^−3^
Δρ_min_ = −1.73 e Å^−3^

### 

Data collection: *MACH3* (Enraf–Nonius, 1993 ▶); cell refinement: *MACH3*; data reduction: *MolEN* (Fair, 1990 ▶); program(s) used to solve structure: *SHELXS97* (Sheldrick, 2008 ▶); program(s) used to refine structure: *SHELXL97* (Sheldrick, 2008 ▶); molecular graphics: *ATOMS* (Dowty, 2002 ▶); software used to prepare material for publication: *publCIF* (Westrip, 2008 ▶).

## Supplementary Material

Crystal structure: contains datablocks I, global. DOI: 10.1107/S1600536808010386/wm2175sup1.cif

Structure factors: contains datablocks I. DOI: 10.1107/S1600536808010386/wm2175Isup2.hkl

Additional supplementary materials:  crystallographic information; 3D view; checkCIF report

## Figures and Tables

**Table 1:** Selected bond lengths (Å)

Pr1—O4	2.370 (3)
Pr1—O3^i^	2.430 (3)
Pr1—O1^i^	2.450 (3)
Pr1—O5^ii^	2.461 (3)
Pr1—O7	2.480 (3)
Pr1—O2	2.529 (3)
Pr1—O2^iii^	2.557 (3)
Pr1—O6^iv^	2.610 (3)
Pr2—O8	2.364 (3)
Pr2—O8^v^	2.375 (3)
Pr2—O4^vi^	2.456 (3)
Pr2—O3	2.506 (3)
Pr2—O1	2.513 (3)
Pr2—O3^vii^	2.585 (3)
Pr2—O1^viii^	2.585 (3)
Pr2—O6^ix^	2.645 (4)
Mo—O5	1.748 (3)
Mo—O7	1.748 (3)
Mo—O6	1.782 (4)
Mo—O2^x^	1.803 (3)
B1—O8	1.345 (6)
B1—O4	1.370 (6)
B1—O9	1.387 (6)
B2—O9^xi^	1.373 (6)
B2—O1^xi^	1.378 (6)
B2—O3	1.384 (6)

Symmetry codes: (i) 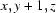; (ii) 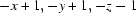; (iii) 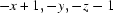; (iv) 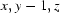; (v) 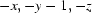; (vi) 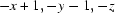; (vii) 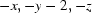; (viii) 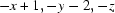; (ix) 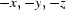; (x) 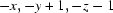; (xi) 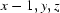.

**Fig. 1.**
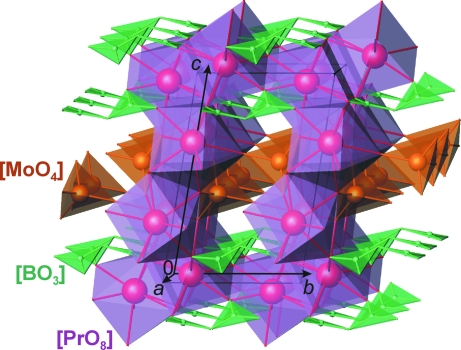
: View of the structure of (I) approximately along the a-axis, emphasizing coordination surroundings of the cations. Pr atoms are shown as large red spheres, Mo atoms as smaller orange spheres, and B atoms as small green spheres. O atoms are indicated by the corners of the coordination polyhedra and are not drawn.

**Fig. 2.**
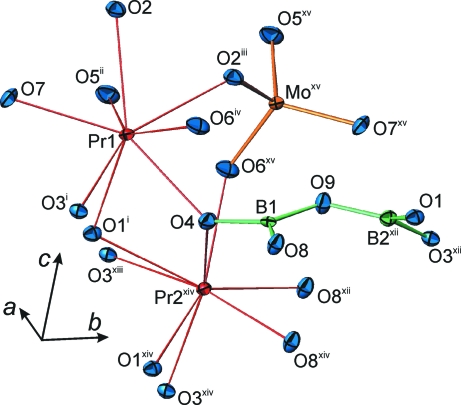
: Fraction of the structure of (I) with atomic labelling scheme in a projection approximately along the a-axis. The atoms are drawn as displacement ellipsoids at the 50% probability level. [Symmetry codes: (i) x, y + 1, z; (ii) -x + 1, -y + 1, -z - 1; (iii) -x + 1, -y, (iv) x, y - 1, z; -z - 1; (x) -x, -y, -z; (xii) x + 1, y, z; (xiii) x + 1, y + 1, z; (xiv) -x + 1, -y - 1, -z; (xv) x + 1, y - 1, z.]

## supplementary crystallographic information

### Comment

The existence of several compounds in the systems *RE*_2_O_3_— B_2_O_3_—MoO_3_ (*RE* = rare earth element) has been studied by Lysanova *et al.* (1983) by means of X-ray powder diffraction data and was partly corroborated by Dzhurinskii & Lysanova (1998). Among these pseudo-ternary compounds, rare earth pyroborate molybdates of the type *RE*_2_(B_2_O_5_)(MoO_4_) were reported for *RE* = Pr - Tb (excluding *Pm*) (Lysanova *et al.*, 1983), all with incongruent melting behaviour. However, no structural information about these compounds has been given so far. In the course of our investigations of the systems *RE*_2_O_3_—B_2_O_3_—*M*O_3_ (*M* = Mo, W) we grew single crystals of the Pr-compound Pr_2_(B_2_O_5_)(MoO_4_), (I), as a representative of the rare earth pyroborate molybdate series.

In the crystal structure of (I) the two symmetrically non-equivalent Pr atoms show a distinct eightfold coordination by oxygen atoms, both with a slightly distorted square antiprismatic coordination polyhedron, and with Pr—O bond lengths ranging from 2.370 (3) Å to 2.610 (3) Å for Pr1, and from 2.364 (3) Å to 2.645 (4) Å for Pr2 (Fig. 2). The [PrO_8_] polyhedra are connected *via* common edges, where Pr2 is connected to six neighbouring [PrO_8_] polyhedra with Pr—Pr distances ranging from 3.9237 (5) Å to 4.1242 (5) Å, while Pr1 is connected to only four [PrO_8_] polyhedra with Pr—Pr distances between 3.9702 (6) Å and 4.1583 (6) Å. From the different connection schemes of the two Pr atoms a three-dimensional framework of [PrO_8_] polyhedra with interstitial voids results (Fig. 1). In these voids nearly undistorted and isolated [MoO_4_] tetrahedra, that are arranged in layers parallel to the *ab* plane are positioned (Fig. 1).

The two crystallographically different B atoms are threefold coordinated by O atoms. The two [BO_3_] groups are linked by a common oxygen ligand O9 (see Fig. 2), thus forming isolated pyroborate groups [B_2_O_5_]. The pyroborate groups are bent with an angle (B1— O9— B2) of 125.1 (4)°, while the individual [BO_3_] groups show no unusual distortions (Zobetz, 1982). The oxygen ligands of the pyroborate group (apart from the bridging oxygen O9, see Fig. 2) each belong either to two different [PrO_8_] polyhedra (being simultaneously ligands of B1) or to three different [PrO_8_] polyhedra (being simultaneously ligands of B2). All [B_2_O_5_] groups are arranged in double layers that extend parallel to the *ab*-plane and alternate with layers of [MoO_4_] tetrahedra (see Fig. 1).

### Experimental

Single crystals of (I) were obtained by growth from the melt. A homogenized powder mixture of Pr_4_O_11_ (99.9%, Alfa Aesar), B_2_O_3_ (99.98%, Alfa Aesar) and MoO_3_ (99.95%, Alfa Aesar) in a molar ratio of 1: 3.33: 7 was heated in a covered platinum crucible in air atmosphere to 1423 K and subsequently cooled at a rate of 3 K h^-1^ to 1173 K. Transparent, light-green prismatic single crystals of the title compound were separated mechanically from the fine-grained praseodymium borate molybdate matrix.

### Refinement

The final difference Fourier map indicated a positive maximum at a distance of 0.76 Å from Pr1 and a negative maximum at a distance of 0.85 Å from the same atom.

### Crystal data

**Table:**

Pr_2_(B_2_O_5_)(MoO_4_)	*Z* = 2
*M**_r_* = 543.38	*F*_000_ = 484
Triclinic, *P*1	*D*_x_ = 5.059 Mg m^−^^3^
*a* = 5.2806 (5) Å	Mo *K*α radiation λ = 0.71073 Å
*b* = 7.0278 (5) Å	Cell parameters from 25 reflections
*c* = 10.5824 (9) Å	θ = 20.8–24.2º
α = 74.557 (6)º	µ = 15.20 mm^−^^1^
β = 76.307 (7)º	*T* = 291 (2) K
γ = 73.065 (6)º	Prism, light green
*V* = 356.69 (5) Å^3^	0.20 × 0.15 × 0.12 mm

### Data collection

**Table:**

Enraf–Nonius CAD-4 diffractometer	*R*_int_ = 0.019
Radiation source: fine-focus sealed tube	θ_max_ = 30.4º
Monochromator: graphite	θ_min_ = 3.1º
*T* = 291(2) K	*h* = −7→7
ω/2θ scans	*k* = −10→10
Absorption correction: ψ scan(MolEN; Fair, 1990)	*l* = −15→15
*T*_min_ = 0.296, *T*_max_ = 0.999	3 standard reflections
4733 measured reflections	every 100 reflections
2155 independent reflections	intensity decay: 1.7%
1983 reflections with *I* > 2σ(*I*)	

### Refinement

**Table:**

Refinement on *F*^2^	Secondary atom site location: difference Fourier map
Least-squares matrix: full	*w* = 1/[σ^2^(*F*_o_^2^) + (0.0533*P*)^2^ + 1.1726*P*] where *P* = (*F*_o_^2^ + 2*F*_c_^2^)/3
*R*[*F*^2^ > 2σ(*F*^2^)] = 0.031	(Δ/σ)_max_ < 0.001
*wR*(*F*^2^) = 0.082	Δρ_max_ = 2.55 e Å^−^^3^
*S* = 1.13	Δρ_min_ = −1.73 e Å^−^^3^
2155 reflections	Extinction correction: SHELXL97 (Sheldrick, 2008), Fc^*^=kFc[1+0.001xFc^2^λ^3^/sin(2θ)]^-1/4^
127 parameters	Extinction coefficient: 0.0319 (13)
Primary atom site location: structure-invariant direct methods	

### Special details

**Table:**

Experimental. A suitable single-crystal was carefully selected under a polarizing microscope and mounted in a glass capillary.
Geometry. All e.s.d.'s (except the e.s.d. in the dihedral angle between two l.s. planes) are estimated using the full covariance matrix. The cell e.s.d.'s are taken into account individually in the estimation of e.s.d.'s in distances, angles and torsion angles; correlations between e.s.d.'s in cell parameters are only used when they are defined by crystal symmetry. An approximate (isotropic) treatment of cell e.s.d.'s is used for estimating e.s.d.'s involving l.s. planes.

### Fractional atomic coordinates and isotropic or equivalent isotropic displacement parameters (Å^2^)

**Table:**

	*x*	*y*	*z*	*U*_iso_*/*U*_eq_	
Pr1	0.37016 (5)	0.07689 (4)	−0.31461 (2)	0.00706 (11)	
Pr2	0.15410 (5)	−0.79427 (4)	0.04666 (2)	0.00693 (11)	
Mo	0.03514 (8)	0.70782 (6)	−0.41958 (4)	0.00758 (12)	
O7	0.1449 (8)	0.4427 (5)	−0.3778 (4)	0.0144 (7)	
O5	0.2335 (7)	0.7903 (6)	−0.5706 (3)	0.0152 (7)	
O6	0.0805 (7)	0.8248 (6)	−0.2991 (4)	0.0141 (7)	
O2	0.3079 (7)	0.1829 (5)	−0.5554 (3)	0.0111 (6)	
B1	0.4573 (10)	−0.4118 (7)	−0.1373 (5)	0.0077 (8)	
O4	0.4775 (7)	−0.2184 (5)	−0.1465 (3)	0.0114 (6)	
O8	0.2350 (7)	−0.4758 (5)	−0.0732 (3)	0.0114 (6)	
O9	0.6870 (7)	−0.5435 (5)	−0.1886 (4)	0.0121 (7)	
B2	−0.2634 (10)	−0.7520 (8)	−0.1570 (5)	0.0091 (8)	
O1	0.5437 (7)	−0.8575 (5)	−0.1392 (3)	0.0094 (6)	
O3	−0.0094 (7)	−0.8664 (5)	−0.1355 (3)	0.0086 (6)	

### Atomic displacement parameters (Å^2^)

**Table:**

	*U*^11^	*U*^22^	*U*^33^	*U*^12^	*U*^13^	*U*^23^
Pr1	0.00931 (15)	0.00715 (15)	0.00456 (15)	−0.00225 (10)	−0.00160 (10)	−0.00032 (10)
Pr2	0.00839 (16)	0.00662 (15)	0.00584 (15)	−0.00215 (10)	−0.00193 (10)	−0.00046 (9)
Mo	0.0094 (2)	0.0072 (2)	0.00587 (19)	−0.00159 (14)	−0.00209 (14)	−0.00082 (13)
O7	0.0190 (18)	0.0082 (15)	0.0112 (15)	−0.0002 (12)	−0.0023 (14)	0.0025 (12)
O5	0.0167 (17)	0.0188 (17)	0.0100 (15)	−0.0069 (14)	0.0023 (13)	−0.0039 (13)
O6	0.0165 (17)	0.0179 (17)	0.0109 (15)	−0.0060 (13)	−0.0036 (13)	−0.0052 (13)
O2	0.0110 (15)	0.0136 (16)	0.0096 (15)	−0.0016 (12)	−0.0034 (12)	−0.0038 (12)
B1	0.011 (2)	0.008 (2)	0.0034 (19)	−0.0022 (16)	−0.0003 (16)	−0.0011 (15)
O4	0.0159 (16)	0.0096 (15)	0.0101 (15)	−0.0046 (12)	−0.0044 (13)	−0.0008 (11)
O8	0.0093 (15)	0.0086 (14)	0.0130 (16)	−0.0015 (11)	0.0004 (13)	0.0005 (12)
O9	0.0120 (16)	0.0075 (15)	0.0142 (17)	−0.0020 (12)	0.0011 (13)	−0.0012 (12)
B2	0.009 (2)	0.009 (2)	0.008	−0.0028 (16)	−0.0004 (17)	−0.0010 (16)
O1	0.0099 (15)	0.0107 (15)	0.0088 (14)	−0.0036 (12)	−0.0030 (12)	−0.0017 (11)
O3	0.0076 (14)	0.0106 (15)	0.0071 (14)	−0.0025 (11)	0.0007 (11)	−0.0024 (11)

### Geometric parameters (Å, °)

**Table:**

Pr1—O4	2.370 (3)	Pr2—O1^ix^	2.585 (3)
Pr1—O3^i^	2.430 (3)	Pr2—O6^x^	2.645 (4)
Pr1—O1^i^	2.450 (3)	Pr2—B2^viii^	3.021 (5)
Pr1—O5^ii^	2.461 (3)	Pr2—Pr2^vii^	3.9237 (6)
Pr1—O7	2.480 (3)	Pr2—Pr1^vi^	3.9702 (5)
Pr1—O2	2.529 (3)	Pr2—Pr1^iv^	3.9893 (5)
Pr1—O2^iii^	2.557 (3)	Mo—O5	1.748 (3)
Pr1—O6^iv^	2.610 (3)	Mo—O7	1.748 (3)
Pr1—Mo^v^	3.6714 (6)	Mo—O6	1.782 (4)
Pr1—Pr2^vi^	3.9702 (5)	Mo—O2^v^	1.803 (3)
Pr1—Pr2^i^	3.9893 (5)	Mo—Pr1^v^	3.6714 (6)
Pr1—Pr2^vii^	4.0171 (5)	B1—O8	1.345 (6)
Pr2—O8	2.364 (3)	B1—O4	1.370 (6)
Pr2—O8^vii^	2.375 (3)	B1—O9	1.387 (6)
Pr2—O4^vi^	2.456 (3)	B2—O9^xi^	1.373 (6)
Pr2—O3	2.506 (3)	B2—O1^xi^	1.378 (6)
Pr2—O1	2.513 (3)	B2—O3	1.384 (6)
Pr2—O3^viii^	2.585 (3)	B2—Pr2^viii^	3.021 (5)
			
O4—Pr1—O3^i^	77.59 (12)	O4^vi^—Pr2—O1^ix^	64.22 (10)
O4—Pr1—O1^i^	67.58 (11)	O3—Pr2—O1^ix^	102.94 (11)
O3^i^—Pr1—O1^i^	73.79 (11)	O1—Pr2—O1^ix^	75.10 (12)
O4—Pr1—O5^ii^	112.06 (12)	O3^viii^—Pr2—O1^ix^	54.11 (11)
O3^i^—Pr1—O5^ii^	139.81 (11)	O8—Pr2—O6^x^	117.87 (11)
O1^i^—Pr1—O5^ii^	74.69 (11)	O8^vii^—Pr2—O6^x^	68.69 (12)
O4—Pr1—O7	149.37 (12)	O4^vi^—Pr2—O6^x^	79.69 (11)
O3^i^—Pr1—O7	75.46 (11)	O3—Pr2—O6^x^	126.73 (10)
O1^i^—Pr1—O7	90.93 (12)	O1—Pr2—O6^x^	154.52 (11)
O5^ii^—Pr1—O7	80.77 (12)	O3^viii^—Pr2—O6^x^	69.74 (10)
O4—Pr1—O2	141.05 (11)	O1^ix^—Pr2—O6^x^	82.67 (11)
O3^i^—Pr1—O2	121.14 (11)	O8—Pr2—Pr2^vii^	34.21 (8)
O1^i^—Pr1—O2	146.59 (11)	O8^vii^—Pr2—Pr2^vii^	34.03 (8)
O5^ii^—Pr1—O2	76.77 (11)	O4^vi^—Pr2—Pr2^vii^	97.53 (8)
O7—Pr1—O2	67.53 (11)	O3—Pr2—Pr2^vii^	93.69 (8)
O4—Pr1—O2^iii^	76.63 (11)	O1—Pr2—Pr2^vii^	103.65 (8)
O3^i^—Pr1—O2^iii^	145.67 (11)	O3^viii^—Pr2—Pr2^vii^	140.80 (7)
O1^i^—Pr1—O2^iii^	115.72 (11)	O1^ix^—Pr2—Pr2^vii^	161.72 (7)
O5^ii^—Pr1—O2^iii^	71.93 (11)	O6^x^—Pr2—Pr2^vii^	93.54 (8)
O7—Pr1—O2^iii^	133.79 (11)	B2^viii^—Pr2—Pr2^vii^	166.65 (10)
O2—Pr1—O2^iii^	70.32 (12)	O8—Pr2—Pr1^vi^	113.11 (9)
O4—Pr1—O6^iv^	69.35 (11)	O8^vii^—Pr2—Pr1^vi^	125.58 (9)
O3^i^—Pr1—O6^iv^	72.69 (11)	O4^vi^—Pr2—Pr1^vi^	33.96 (8)
O1^i^—Pr1—O6^iv^	129.69 (11)	O3—Pr2—Pr1^vi^	139.70 (8)
O5^ii^—Pr1—O6^iv^	147.48 (12)	O1—Pr2—Pr1^vi^	90.73 (8)
O7—Pr1—O6^iv^	115.17 (12)	O3^viii^—Pr2—Pr1^vi^	79.17 (7)
O2—Pr1—O6^iv^	83.51 (11)	O1^ix^—Pr2—Pr1^vi^	36.78 (7)
O2^iii^—Pr1—O6^iv^	77.21 (11)	O6^x^—Pr2—Pr1^vi^	63.84 (8)
O4—Pr1—Pr2^vi^	35.36 (8)	B2^viii^—Pr2—Pr1^vi^	57.28 (10)
O3^i^—Pr1—Pr2^vi^	89.53 (8)	Pr2^vii^—Pr2—Pr1^vi^	126.063 (11)
O1^i^—Pr1—Pr2^vi^	39.18 (8)	O8—Pr2—Pr1^iv^	82.46 (9)
O5^ii^—Pr1—Pr2^vi^	81.40 (9)	O8^vii^—Pr2—Pr1^iv^	115.15 (9)
O7—Pr1—Pr2^vi^	129.95 (9)	O4^vi^—Pr2—Pr1^iv^	115.41 (8)
O2—Pr1—Pr2^vi^	149.14 (8)	O3—Pr2—Pr1^iv^	35.45 (7)
O2^iii^—Pr1—Pr2^vi^	82.37 (8)	O1—Pr2—Pr1^iv^	35.98 (7)
O6^iv^—Pr1—Pr2^vi^	104.66 (8)	O3^viii^—Pr2—Pr1^iv^	88.62 (7)
Mo^v^—Pr1—Pr2^vi^	173.822 (12)	O1^ix^—Pr2—Pr1^iv^	89.26 (7)
O4—Pr1—Pr2^i^	68.51 (8)	O6^x^—Pr2—Pr1^iv^	157.63 (7)
O3^i^—Pr1—Pr2^i^	36.74 (8)	B2^viii^—Pr2—Pr1^iv^	87.02 (10)
O1^i^—Pr1—Pr2^i^	37.06 (8)	Pr2^vii^—Pr2—Pr1^iv^	100.262 (11)
O5^ii^—Pr1—Pr2^i^	108.19 (8)	Pr1^vi^—Pr2—Pr1^iv^	118.956 (9)
O7—Pr1—Pr2^i^	81.16 (8)	O5—Mo—O7	107.10 (17)
O2—Pr1—Pr2^i^	147.30 (8)	O5—Mo—O6	107.70 (16)
O2^iii^—Pr1—Pr2^i^	142.36 (8)	O7—Mo—O6	111.77 (17)
O6^iv^—Pr1—Pr2^i^	102.41 (8)	O5—Mo—O2^v^	105.62 (17)
Mo^v^—Pr1—Pr2^i^	124.637 (12)	O7—Mo—O2^v^	118.20 (16)
Pr2^vi^—Pr1—Pr2^i^	61.044 (9)	O6—Mo—O2^v^	105.89 (16)
O4—Pr1—Pr2^vii^	53.61 (8)	Pr1^i^—O6—Pr2^x^	99.70 (12)
O3^i^—Pr1—Pr2^vii^	38.13 (7)	Mo^v^—O2—Pr1	114.85 (15)
O1^i^—Pr1—Pr2^vii^	91.90 (8)	Mo^v^—O2—Pr1^iii^	122.00 (16)
O5^ii^—Pr1—Pr2^vii^	164.08 (9)	Pr1—O2—Pr1^iii^	109.68 (12)
O7—Pr1—Pr2^vii^	108.49 (8)	O8—B1—O4	122.1 (4)
O2—Pr1—Pr2^vii^	118.48 (8)	O8—B1—O9	121.5 (4)
O2^iii^—Pr1—Pr2^vii^	107.57 (8)	O4—B1—O9	116.2 (4)
O6^iv^—Pr1—Pr2^vii^	40.47 (8)	B1—O4—Pr1	129.0 (3)
Mo^v^—Pr1—Pr2^vii^	97.798 (12)	B1—O4—Pr2^vi^	113.9 (3)
Pr2^vi^—Pr1—Pr2^vii^	82.769 (10)	Pr1—O4—Pr2^vi^	110.68 (12)
Pr2^i^—Pr1—Pr2^vii^	62.010 (9)	B1—O8—Pr2	134.1 (3)
O8—Pr2—O8^vii^	68.24 (13)	B1—O8—Pr2^vii^	113.4 (3)
O8—Pr2—O4^vi^	79.16 (11)	Pr2—O8—Pr2^vii^	111.76 (13)
O8^vii^—Pr2—O4^vi^	113.81 (11)	B2^xii^—O9—B1	125.1 (4)
O8—Pr2—O3	95.79 (11)	O9^xi^—B2—O1^xi^	123.8 (4)
O8^vii^—Pr2—O3	90.33 (12)	O9^xi^—B2—O3	119.3 (4)
O4^vi^—Pr2—O3	150.59 (11)	O1^xi^—B2—O3	116.8 (4)
O8—Pr2—O1	72.10 (11)	Pr1^iv^—O1—Pr2	106.97 (12)
O8^vii^—Pr2—O1	134.09 (11)	B2^xii^—O1—Pr2^ix^	94.4 (3)
O4^vi^—Pr2—O1	79.52 (11)	Pr1^iv^—O1—Pr2^ix^	104.05 (12)
O3—Pr2—O1	71.43 (11)	Pr2—O1—Pr2^ix^	104.90 (12)
O8—Pr2—O3^viii^	167.27 (12)	B2—O3—Pr1^iv^	123.2 (3)
O8^vii^—Pr2—O3^viii^	108.01 (11)	B2—O3—Pr2	114.9 (3)
O4^vi^—Pr2—O3^viii^	113.02 (11)	Pr1^iv^—O3—Pr2	107.81 (12)
O3—Pr2—O3^viii^	71.81 (12)	B2—O3—Pr2^viii^	94.2 (3)
O1—Pr2—O3^viii^	105.54 (10)	Pr1^iv^—O3—Pr2^viii^	106.39 (12)
O8—Pr2—O1^ix^	134.34 (11)	Pr2—O3—Pr2^viii^	108.19 (12)
O8^vii^—Pr2—O1^ix^	150.80 (11)		

Symmetry codes: (i) *x*, *y*+1, *z*; (ii) −*x*+1, −*y*+1, −*z*−1; (iii) −*x*+1, −*y*, −*z*−1; (iv) *x*, *y*−1, *z*; (v) −*x*, −*y*+1, −*z*−1; (vi) −*x*+1, −*y*−1, −*z*; (vii) −*x*, −*y*−1, −*z*; (viii) −*x*, −*y*−2, −*z*; (ix) −*x*+1, −*y*−2, −*z*; (x) −*x*, −*y*, −*z*; (xi) *x*−1, *y*, *z*; (xii) *x*+1, *y*, *z*.
